# Unlocking Opportunities for *Mycobacterium leprae* and *Mycobacterium ulcerans*

**DOI:** 10.1021/acsinfecdis.3c00371

**Published:** 2024-01-31

**Authors:** Mousumi Shyam, Sumit Kumar, Vinayak Singh

**Affiliations:** † Department of Pharmaceutical Sciences & Technology, Birla Institute of Technology, Mersa, Ranchi, Jharkhand 835215, India; ‡ Holistic Drug Discovery and Development (H3D) Centre, University of Cape Town, Rondebosch 7701, South Africa; § South African Medical Research Council Drug Discovery and Development Research Unit, University of Cape Town, Rondebosch 7701, South Africa; @ Institute of Infectious Disease and Molecular Medicine (IDM), University of Cape Town, Observatory 7925, Cape Town, South Africa

**Keywords:** *Mycobacterium ulcerans*, *Mycobacterium leprae*, Buruli ulcer, leprosy, mycolactone biosynthesis, mycolactone analogues, drug repurposing, PKS inhibitors, 3M mechanisms, efflux pump inhibitors, antituberculars

## Abstract

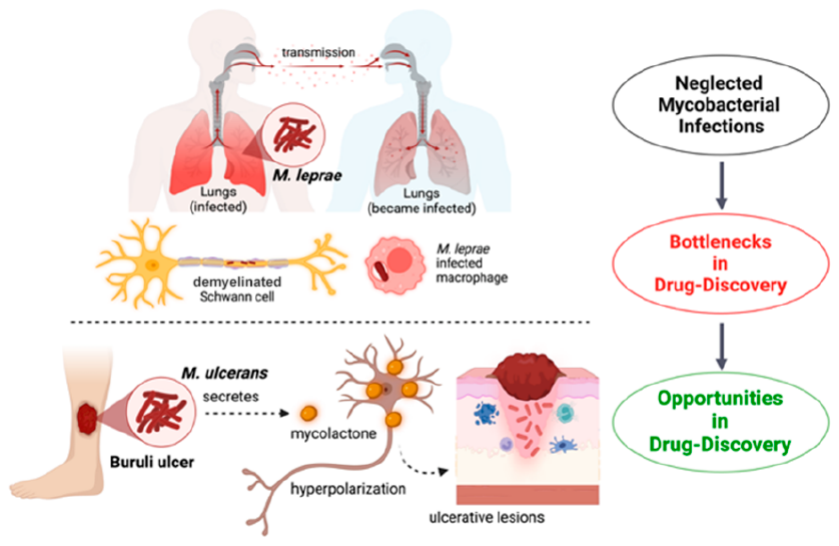

In the recent decade, scientific communities have toiled to tackle the emerging burden of drug-resistant tuberculosis (DR-TB) and rapidly growing opportunistic nontuberculous mycobacteria (NTM). Among these, two neglected mycobacteria species of the Acinetobacter family, *Mycobacterium leprae* and *Mycobacterium ulcerans*, are the etiological agents of leprosy and Buruli ulcer infections, respectively, and fall under the broad umbrella of neglected tropical diseases (NTDs). Unfortunately, lackluster drug discovery efforts have been made against these pathogenic bacteria in the recent decade, resulting in the discovery of only a few countable hits and majorly repurposing anti-TB drug candidates such as telacebec (Q203), P218, and TB47 for current therapeutic interventions. Major ignorance in drug candidate identification might aggravate the dramatic consequences of rapidly spreading mycobacterial NTDs in the coming days. Therefore, this Review focuses on an up-to-date account of drug discovery efforts targeting selected druggable targets from both bacilli, including the accompanying challenges that have been identified and are responsible for the slow drug discovery. Furthermore, a succinct discussion of the all-new possibilities that could be alternative solutions to mitigate the neglected mycobacterial NTD burden and subsequently accelerate the drug discovery effort is also included. We anticipate that the state-of-the-art strategies discussed here may attract major attention from the scientific community to navigate and expand the roadmap for the discovery of next-generation therapeutics against these NTDs.

The World Health Organization (WHO) has listed Hansen’s disease (HD) and Buruli ulcer (BU) as the second and third most common mycobacterial diseases, respectively, after the deadliest infectious disease, tuberculosis (TB).^1^ HD and BU are primarily caused by the obligate intracellular bacilli *Mycobacterium leprae* and *Mycobacterium ulcerans*.^2^ Research suggests that the recently discovered *Mycobacterium lepromatosis*, which is linked to the bacillus *M. leprae*, also contributes to the spread of leprosy in some regions.^2,3^ HD, also termed “lepromatous leprosy”, is a chronic granulomatous disease that continues to be endemic in many parts of the world (mostly observed in tropical and subtropical regions, e.g., India, Indonesia, and Brazil).^4,5^ Individuals with HD or commonly known leprosy are clinically identified as having skin lesions and peripheral nerve damage, which subsequently result in the loss of sensation, paralysis, and deformities. Unlike most other infectious diseases, the mode of transmission for leprosy is still up for discussion, though most leprologists would like to support that infection is airborne by way of the upper respiratory tract, which has been supported by the evidence of the abundance of *M. leprae* bacilli in the nasal discharge of leprosy patients (Figure 1).^6,7^

**Figure 1:**
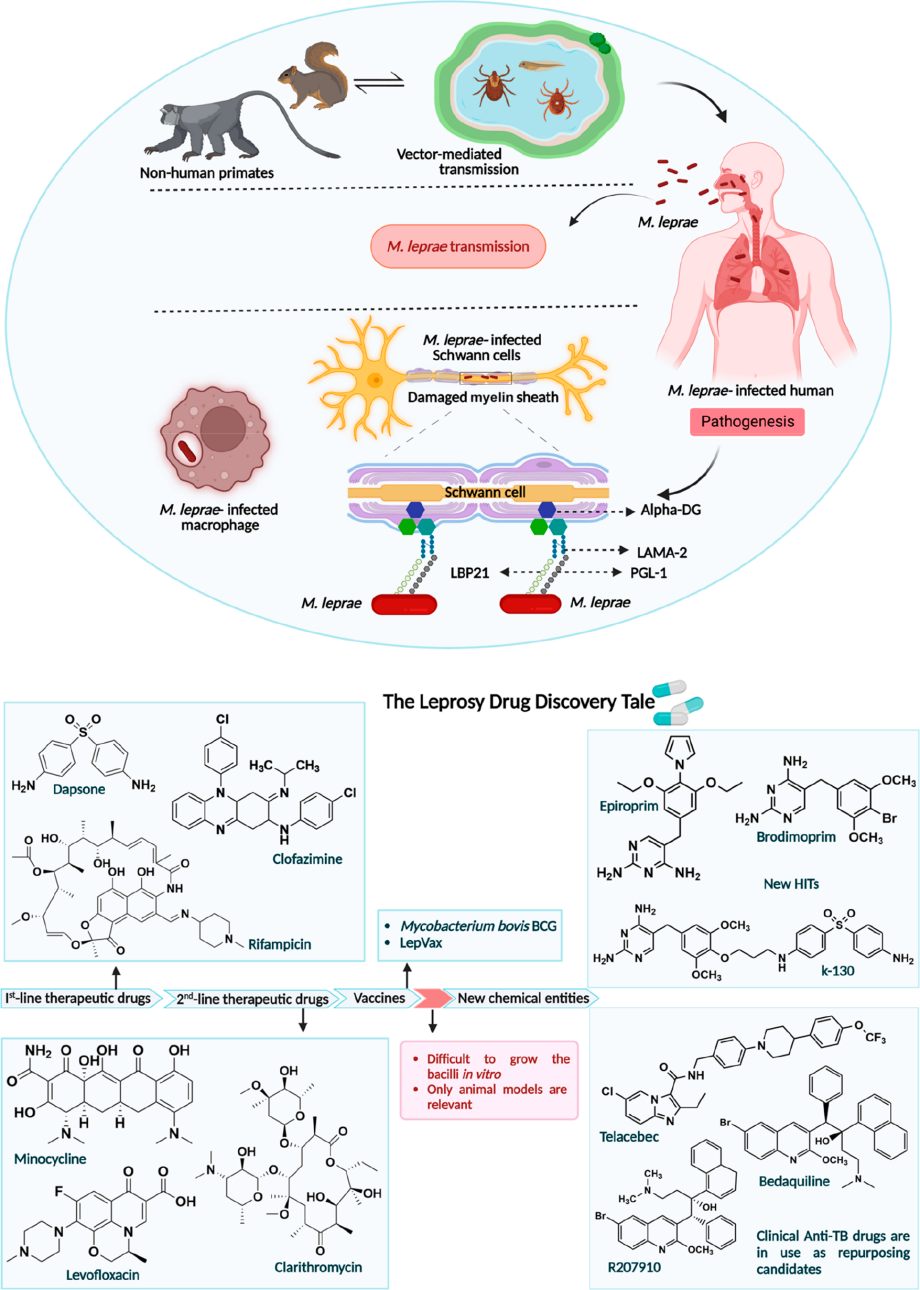
*Mycobacterium leprae* infection cycle and a summary of antileprosy drug discovery. Phenolic glycolipid I (PGL-I) and laminin binding protein 21 (LBP21) are the surface antigens present on the cell wall of *M. leprae*. These surface antigens are involved in the *M. leprae* invasion of Schwann cells through binding to the α-dystroglycan (Alpha-DG) and α-2 chain of the laminin-2 (LAMA2) at the basal lamina. The major function of the Schwann cells is to synthesize the myelin sheath, and when these cells are infected by *M. leprae* as a consequence, this results in demyelination and causes peripheral nerve damage.

On the other hand, BU is recognized as one of the skin-related neglected tropical diseases (NTDs). The disease BU was named after the Buruli county in Uganda in Africa, where a large number of cases were first reported in the 1960s.^8^ BU is primarily characterized by nodules, plaques, or edematous lesions that eventually progress to extensive indolent necrotizing skin ulceration.^9,10^ Interestingly, the pathogen *M. ulcerans* is closely related to a particular nontuberculous mycobacteria (NTM) species, i.e., *Mycobacterium marinum*, and similarly, this slow-growing pathogen secretes a toxic polyketide called mycolactone, which triggers inflammation. Mycolactone is the virulent factor of *M. ulcerans* and performs cytotoxic activity to induce apoptosis followed by necrosis of several cell types, such as adipocytes and fibroblasts, at the infection foci. Therefore, perhaps, mycolactone can be considered as an immunomodulator and cytotoxin (Figure 2).^11−14^ BU is prevalent in the rural areas of tropical and subtropical regions with a high focal distribution along the waterbodies such as Asia, Africa, Australia, and America. In the past decade, more than 20 000 cases were reported in West Africa alone, a record number with the highest prevalence rates. In particular, Ghana, Nigeria, and Benin are leading the prevalence rates.^15−17^ Similar to *M. leprae*, the pathogen’s route of transmission is very poorly understood. It is speculated that *M. ulcerans* enters the body through tiny skin incisions after contact with polluted water, soil, or plants or that it may be spread by the bites from aquatic insects.^18,19^

**Figure 2:**
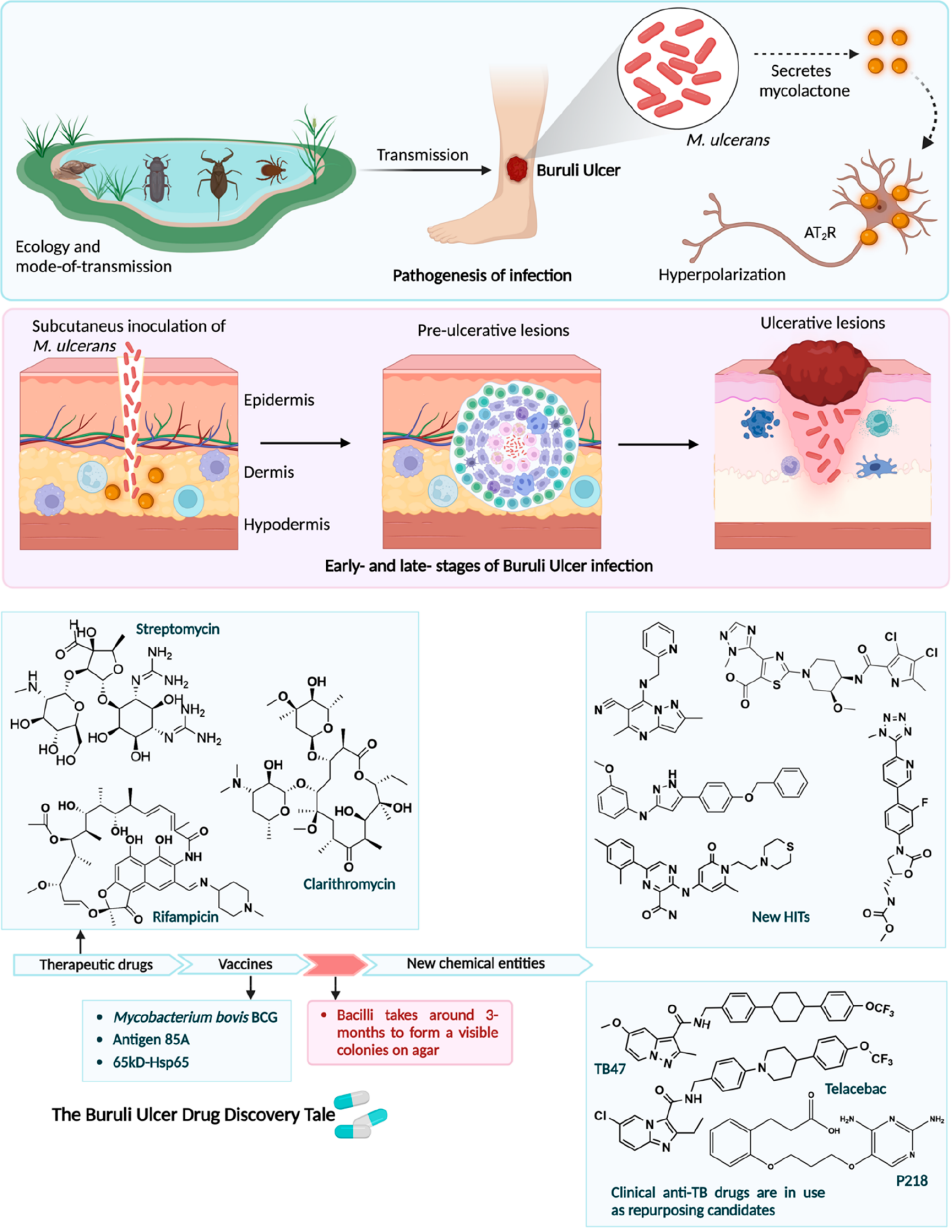
*Mycobacterium ulcerans* transmission and pathogenesis, and a summary of drug discovery efforts. *M. ulcerans* secretes mycolactone, which is a lipid-like toxin and the major virulence factor and etiological agent of Buruli ulcer. Mycolactone elicits signaling through type 2 angiotensin II receptors (AT2Rs), leading to the potassium-dependent hyperpolarization of neurons. Buruli ulcer is characterized by necrotizing skin and soft tissues.

Despite the growing interest in mycobacterial NTDs, many national public health programs have largely overlooked these infections for a long time. Due to the limited knowledge of these pathogens, there is an underestimation of the true disease burden. The concern of severely limited financial resources for innovative drug discovery and development is thus immediately solved by repurposing TB medications, as the prototypical intracellular pathogens *M. leprae* and *M. ulcerans* are both very closely related to *Mycobacterium tuberculosis*; henceforth, drug molecular targets could be conserved between these human pathogenic species.^20−23^ Albeit in the long term, rational drug discovery efforts are needed for these neglected mycobacteria, a sizable number of scientific ideas have been considered in the search for newer solutions for these understudied NTDs. It has been anticipated that more knowledge and a better understanding of these two NTDs will considerably strengthen efforts to combat them. To address the increasing prevalence of mycobacterial NTDs, one must first be well-informed about the initiatives that have already been implemented in the past. Then, one can foresee the cutting-edge, state-of-the-art strategies that could be employed in the foreseeable future.

## Antileprosy Drug Discovery: Progress and Challenges

### How It Started

A comprehensive review of the systematic drug development process for leprosy showed that dapsone was first introduced as the standard chemotherapy for leprosy in the early 1950s (Figure 1). The major drawback associated with bacteriostatic dapsone was that it was required for a long-term, often lifelong, defense against *M. leprae*. This resulted in the emergence of dapsone-resistant strains of *M. leprae* in the 1970s.^24,25^ To mitigate this, two other antimycobacterial drugs, rifampicin and clofazimine, were introduced between the 1960s and 1970s. Importantly, the statistics indicate that rifampicin was a successful antileprosy chemotherapeutic, though using it as a single drug of choice resulted in relapse of leprosy.^25^ Interestingly, clofazimine monotherapy was ineffective due to its weak bactericidal activity against *M. leprae*.^26,27^ To improve treatment effectiveness and avoid the public health crisis created by the widespread use of first-line medications (dapsone, rifampicin, and clofazimine), the WHO suggested multidrug therapy (MDT) for leprosy in 1982. The WHO proposed paucibacillary (PB) leprosy treatment with dapsone and rifampicin for 6 to 12 months and, subsequently, the inclusion of clofazimine in the therapeutic regimen for up to 24 months for the treatment of multibacillary (MB) leprosy. However, it took over 15 years for MDT to be used globally, and the target of fully covering all leprosy patients by 1997 was not achieved.^28^ Nonetheless, the global adoption of MDT was a success story that led to a 50-fold reduction in the number of leprosy patients. While MDT techniques have been effective in treating leprosy, many patients are still coping with long-term sequelae such as irreversible nerve function deficits and disabilities, and in certain cases, acute inflammatory responses are evident (Type I and Type II reactions),^29^ which speed up nerve degeneration.

Dapsone, rifampicin, and clofazimine were widely used as first-line drugs in MDT, but there was also a surge for new drugs due to the limited bactericidal chemotherapeutic availability in the antibiotic regimen and, most importantly, the need to provide a second-line treatment for cases with rifampicin-resistant *M. leprae* strains. Clarithromycin (macrolide), minocycline (tetracycline), and levofloxacin/ofloxacin (fluoroquinolone) are more widely used as second-line therapies due to their high bactericidal activity against *M. leprae* (albeit inferior to rifampicin) in vivo and in clinical studies with fewer side effects (Figure 1).^30,31^ However, after 1997, a resurgence of fluoroquinolone-resistant *M. leprae* strains, particularly those that were already resistant to dapsone and rifampicin, was reported. A remarkable 208 641 new cases were recorded globally in 2019, although the annual incidence has plateaued at above 200 000. According to the WHO, the 23 nations designated as global priorities for the geographical distribution of leprosy account for 95% of all new cases. In addition, the increasing prevalence of multidrug-resistant *M. leprae* in various regions of the world stresses the call to develop new bactericidal chemotypes to expedite effective treatment and lessen the spread of zoonotic transmission.^32,33^

### Major Challenges

The understanding and knowledge of the molecular mechanisms involved in the pathology of various mycobacterial diseases have sped up the discovery of novel therapeutic options. But unlike other mycobacteria, the molecular mechanisms of *M. leprae* are poorly understood because of the difficulties of in vitro growth, and the knowledge is mainly based on animal models (Figure 1). *M. leprae* has a 3.27 Mb genome and a single circular chromosome; comparative genomic analysis has revealed significant variations between it and other mycobacteria. The genome of *M. leprae* only has 49.5% protein-coding genes, and the rest are pseudogenes. Infections are thought to have undergone reductive evolution, which has resulted in the loss of roughly 2000 genes.^34^ Most surprisingly, even though the full genome of *M. leprae* was revealed just over three years after that of *M. tuberculosis,* only 16 X-ray crystallography structures of particular proteins have been deposited in the RCSB protein data bank, or PDB, compared to the 1200 crystallography structures of *M. tuberculosis* proteins that have been deposited.^35−37^

### New Chemical Entities

**Figure 3:**
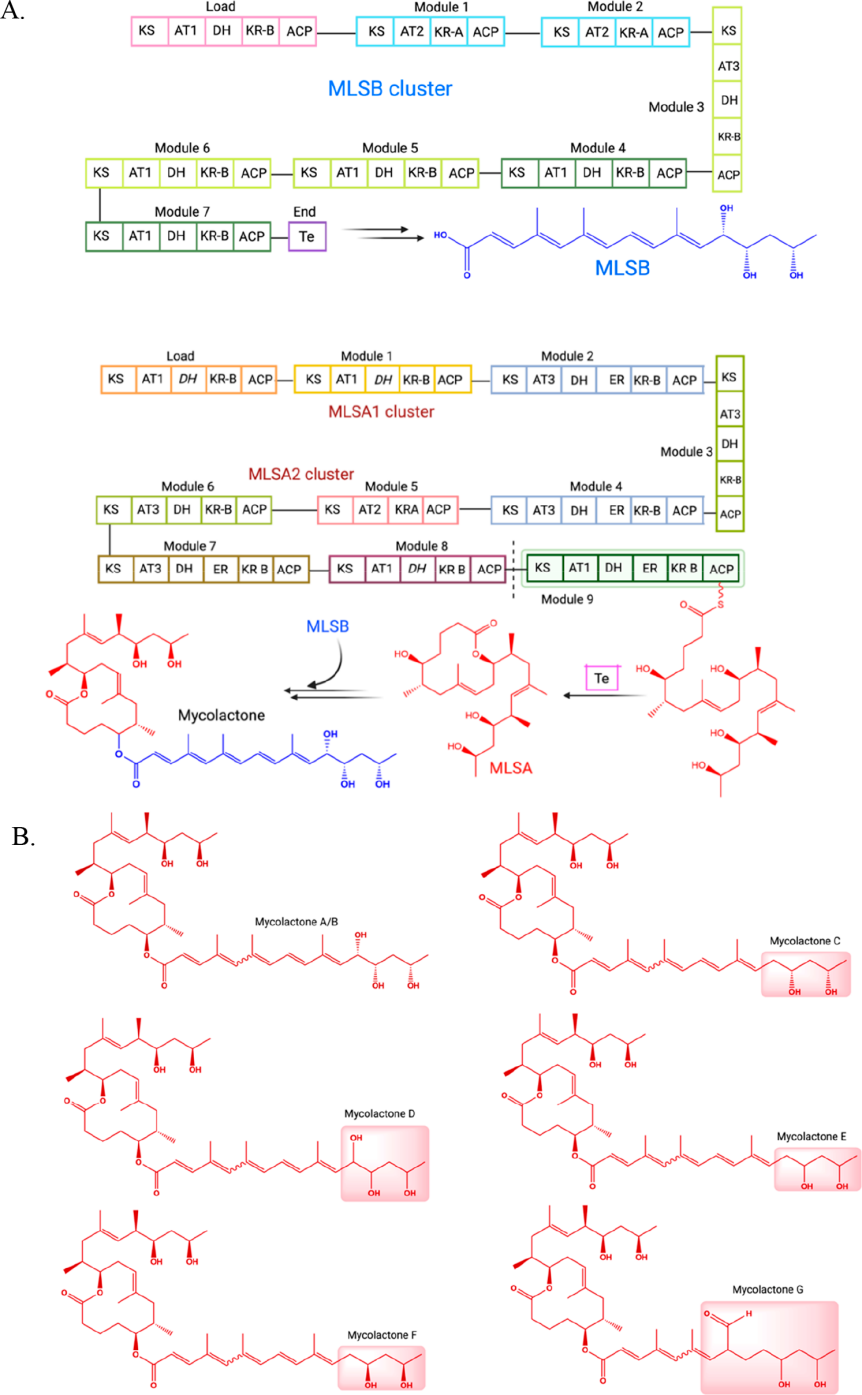
Overview of mycolactone biosynthesis. (A) Gene, module, and domain organization of *mlsB*, *mlsA1*, and *mlsA2* clusters mediated. (B) Chemical structure of mycolactone analogues (mycolactones A–G); the major structural differences are highlighted.

The dihydrofolate reductase (DHFR) inhibitor epiroprim was used in combination with the first-line medication dapsone in a mouse footpad infection model in 2002 (Figure 3).^38^ Epiroprim was extremely effective against both dapsone-sensitive and dapsone-resistant *M. leprae* strains. Earlier, K-130 (2,4-diaminodiphenyl sulfone-substituted 2,4-diamino-5-benzylpyrimidine) and brodimoprim of the same class were used in combination with dapsone and showed cidality against dapsone-resistant *M. leprae* in the mouse foot-pad model. Additionally, both candidates have shown considerable synergy with dapsone (Figure 1).^39,40^

Telacebec (Q203) was discovered as the inhibitor of cytochrome *bcc*:*aa3* terminal oxidase or the cytochrome *bc1* complex cytochrome *b* subunit (QcrB).^41,6^*M. leprae* exclusively relies on QcrB for respiration, and the absence of a QcrB homologue in the human host makes it a lucrative mycobacterial drug target. Telacebec appeared as a promising inhibitor of *M. leprae* at 2.0 nM.^42^ A mouse bone-marrow-derived macrophage model has similarly shown considerable activity against intracellular *M. leprae* at 2.0 nM (Figure 1).

Bedaquiline (BDQ, TMC207), an ATP synthase inhibitor, was developed to treat multidrug-resistant TB.^43^ In a mouse infection model, BDQ has demonstrated promising bactericidal activity against *M. leprae,* equivalent to rifampicin, rifapentine, and moxifloxacin and notably greater than PA-824, linezolid, and minocycline.^44,45^ Favorably, testing of the safety and efficacy of BDQ in MB leprosy patients began in 2018, and it is anticipated that the data will be available by 2024. Importantly, BDQ will be the first clinically approved candidate to have completed a leprosy clinical trial^46^ (Figure 1).

In a mouse footpad infection model, the diarylquinoline candidate R207910 was discovered to be a potential choice for *M. leprae* bactericidal therapy. Like rifampicin, rifapentine, and moxifloxacin in effectiveness, R207910 was substantially more active than PA-824, linezolid, and minocycline.^44^ These findings encourage further exploration of R207910 as a viable leprosy intervention (Figure 1).

Other than the therapeutic candidates, earlier in 1939, the bacille Calmette–Guérin (BCG) vaccine was first proposed against leprosy infection due to possible common antigens between *M. leprae* and *Mycobacterium bovis*.^47,48^ Several studies over the decades have reported promising protection (26–96%) of BCG against leprosy, and in 2018, WHO officially included the single-dose BCG vaccination recommendation for leprosy.^49−51^ Recently, a protein vaccine candidate called LepVax was discovered for the treatment of leprosy infection. Its promising clinical trial findings in healthy subjects encourage its advancement to testing in leprosy endemic regions (Figure 1).^52^ LepVax is a cocktail of ML2055, ML2380, and ML2028 antigens and has shown a better pharmacological profile than the established TB vaccine BCG and other replicating live vaccines, indicating a safer administration to both immunocompetent and immunocompromised people.^53^

## Combating Buruli Ulcer Infection

### Drug and Vaccine Discoveries: How It Started

Treatment of BU is difficult and frequently involves both extensive antibiotic regimens and surgery, which may even require skin transplants. The WHO initially suggested rifampicin with streptomycin or clarithromycin as a two-month course for BU treatment. This treatment requires daily injections of streptomycin for two months, which results in hearing loss, nausea, and vomiting. Therefore, the development of potential biomarkers to monitor a patient’s response to therapy and the introduction of new drugs to combat this infection are urgently required.^54^ Contrary to widespread assumption, there is currently no vaccine against *M. ulcerans*; nevertheless, vaccination with the BCG vaccine has been linked to significant, albeit transient, protection against BU.^55^ Potential possibilities for vaccine development include modified BCG vaccines and immunizations based on the individual subunits.^56^ Previous strategies on using MUL_2232, MUL_3720, Hsp18, and MUL_3720 proteins as prospective vaccine candidates failed to protect against *M. ulcerans* infection in mice despite inducing high antibody titers in immunized mice.^57,58^ However, DNA vaccines encoding the antigen 85A and the 65-kD heat-shock protein Hsp65 could shield mice from *M. ulcerans* infection.^59−61^ However, these were not as protective in mice as BCG, even when administered during DNA prime-boost methods. Unprecedented potential for vaccine development and improved precision medicine could be provided by comparative genomics (Figure 2).

### Major Challenges

The real concern with *M. ulcerans* is the extremely slow growth; it takes around three months for a visible colony to appear on an agar plate that has been supplemented with enrichment media intended for mycobacterial development. Preclinical studies, required for the development of anti-BU chemotherapeutics, would take much longer using conventional methods that count the colony forming units (CFU) to determine a drug’s activity.^62^ Determination of CFU is considered a key readout in the microbiology and immunology domain. However, to address the major time challenge associated with *M. ulcerans*, further focus on the autoluminescent reporter *M. ulcerans* strain (AlMu) as a potential alternative approach is discussed here. AlMu was created by using the *lux*CDABE operon from *Photorhabdus luminescens*. Relative light units (RLU), which are quantified using autoluminescent reporter strains, exhibit a positive connection with CFU counting through conventional approaches. On the other hand, the real-time investigation of in vitro (two days) and in vivo (within a week) drug activity by using recombinant bioluminescent reporter *M. ulcerans* strains, which express *luxAB* genes from *Vibrio harveyi*, was encouraging to expedite the new discoveries within the shorter regimen.^19,63^ In summary, the adoption of recombinant bioluminescent reporter *M. ulcerans* strains made it possible to monitor the pathogen quickly and repeatedly in real-time in a mouse foot infection model to assess the compound’s antimycobacterial activity by greatly reducing the required time, effort, and resources.

### New Chemical Entities

A library of compounds covering a broad chemical space was selected from a TB drug development program of AstraZeneca to screen against *M. ulcerans*. The screening identified five potent candidates with a minimum inhibitory concentration (MIC) of ≤1 μM, indicating a good starting point for the lead development. The chemical structures suggest that fundamental chemical blocks of heterocyclic rings such as pyrazole, thiazole, and imidazole in the scaffold architecture are noticeably efficient against this extremely slow-growing pathogen.^64^ Furthermore, drug design approaches in alignment with this fundamental scaffold layout might be encouraging for inhibitor discovery against BU (Figure 2).

P218 is a derivative of the diaminopyridine candidate WR99210, also known as a dihydrofolate reductase (DHFR) inhibitor, which is now being tested in clinical trials for the treatment of malaria. It has since been discovered to be a powerful inhibitor of the MulDHFR enzyme in *M. ulcerans* in an enzyme inhibition assay. P218 showed a significant enzyme inhibition profile against MulDHFR, while an investigation against *M. ulcerans* in microbiological assays has yet to be conducted, which must be done before claiming its potentiality for infection prevention against BU. The amalgamation of in vitro and in vivo instigations against the pathogen in parallel to drug target identification and validation is required for prospective drug development. The encouraging enzyme inhibition finding raises the possibility that P218 could be explored further for antimycobacterial investigation against *M. ulcerans*. Subsequently, it can be used in combination with other antimicrobial drugs as a more modern means of avoiding the severe adverse effects of rifampicin in the future (Figure 2).^65^

Both in vivo and in vitro testing revealed that the pyrazolo[1,5-*a*]pyridine-3-carboxamide TB47 was highly bactericidal against *M. ulcerans*. When compared to the conventional BU treatment regimen advised by the WHO, TB47 reduces the pathogen’s burden by more than 2.5 log_10_ CFU in a mouse footpad BU infection model. Although it has been recognized that TB47 resistance is conferred by mutations in ubiquinol-cytochrome *c* reductase cytochrome subunit *b*, this drug’s robust pharmacological properties and low level of toxicity call for continued investigation of this treatment for BU (Figure 2).^66^

As a chemotherapy for the treatment of BU, the antitubercular drug telacebec (Q203) is a novel first-in imidazopyridine amide chemical class and exhibits outstanding promise.^6^ Given twice weekly in combination with either bedaquiline or rifapentine, telacebec sterilized mouse footpads in 8 weeks, that is, after a total of just 16 doses, and prevented recurrence for 20 weeks following the conclusion of treatment. These outcomes are extremely encouraging for future intermittent oral regimens that would considerably streamline the field of BU treatment.^67,68^ As a newer hope for tackling the drug-resistance, it has been postulated that Q203 and another imidazopyridine amide may support single-dose combination treatments in the near future (Figure 2).^69^

### Future Directions: Interlinking Drug Repurposing and Alternative Mechanisms As Prospective Treatment Solutions

#### Deciphering Mycolactone Biosynthesis Pathway for Newer Possibilities

Mycolactone is one of the major virulent factors of *M. ulcerans*, characterized by a 12-membered macrolactone core appended to a highly unsaturated acyl side chain.^70,71^ Mycolactone biosynthesis is encoded by the type 1 polyketide synthases (PKS or *mls* cluster) (Figure 3),^72^ comprising three unusually large and homologous gene machineries (*mlsA1*: 51 kb, *mlsB*: 42 kb, and *mlsA2*: 7 kb) with the putative accessory enzymes encoded by a P450 monooxygenase *mup045* and a FabH-like type III ketosynthase *mup038* within the mycobacterial cell wall. The mycolactone PKS orchestra covers the range of diverse enzymes in the assembly line enzymes, i.e., ketosynthase (KS), acyltransferase 1 (AT1), acyltransferase 2 (AT2), acyltransferase 3 (AT3), dehydratase (DH), dehydratase inactive, enoylreductase (ER), ketoreductase A (KR-A), ketoreductase B (KR-B), acyl carrier protein (ACP), intermodular linker, and integral thioesterase (TE). Multi-enzyme PKS machinery produces bioengineered small molecules via iterative Claisen condensation mechanisms by genetically engineered rearrangements of the *mls* mega-module architecture. Each module (M1–M9 of *mlsA1*/*A2* and M1–M7 of *mlsB*) comprises a different set of enzymes that produce instinct intermediate products in the bioengineered mycolactone scaffold.^73^ The *mlsA1* and *mlsA2* clusters combined constitute a loading module along with nine extension modules that synthesize the mycolactone core. On the upper side chain, *mlsB*, along with its loading module and seven extension modules, generates the acyl side chain of the mycolactone architecture.^74,75^

#### Mycolactone and Its Structural Analogues

**Figure 4:**
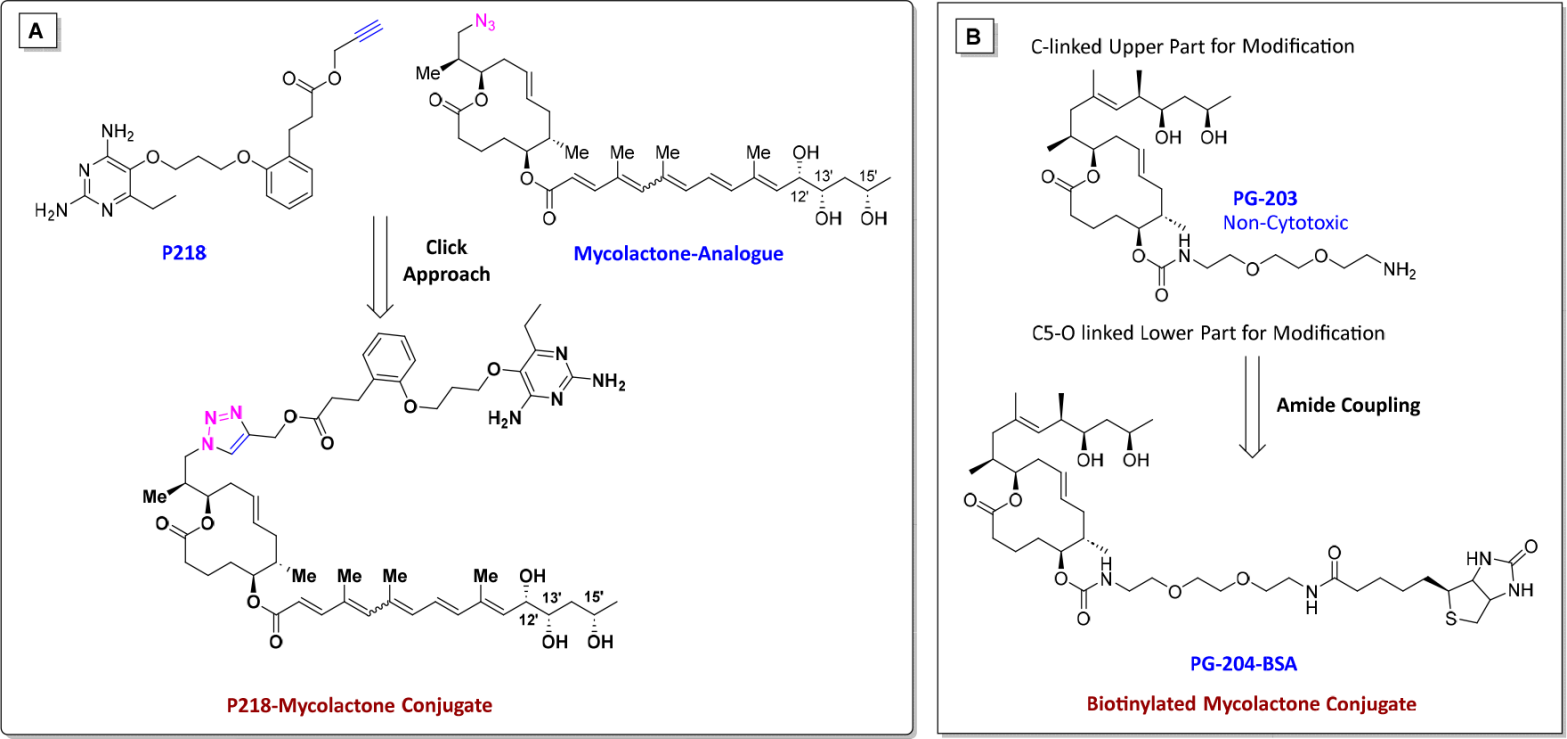
Mycolactone and its structural analogues. (A) A “Trojan Horse” approach for P218-mycolactone conjugate discovery is highlighted here with a possible chemical synthesis strategy using click chemistry. Click approach: the copper-catalyzed azide–alkyne cycloaddition (CuAAC) reaction can be used to achieve targeted conjugates. (B) PG-203, a noncytotoxic synthetic mycolactone derivative, can be coupled using acid-amine coupling reactions (amide coupling) to provide a biotinylated mycolactone conjugate (PG-204-BSA).

It has been speculated that the modules are interchangeable in mycolactone PKS because the domains are of near-identical sequence, supporting that they might be readily exchanged with each other to produce new module combinations, resulting in the development of different mycolactone polyketides (mycolactones C–G^76,77^ in Figure 3) secreted by *Mycobacterium liflandii* and *Mycobacterium pseudoshottsii*.^78^ In the PKS multimodular system, where interdomain identity is less than 80% and tight specificity is accountable for native incoming precursor polyketides for a particular given module and under the same chemical layout, swapping, deleting, or duplicating a particular module resulted in six diverse mycolactone secretion.^79^ In the context of discovering potential treatment options against *M. ulcerans* infections, Pluschke et al. reported a few analogues of synthetic mycolactone A/B, varying both the C-linked upper parts and the C5–O linked lower parts of the core utilizing the stereoselective olefin metathesis ring closure approach. These analogues were evaluated for their cytotoxic potential against murine L929 fibroblasts cell. Further, they synthesized the mycolactone derivative PG-203 (noncytotoxic), which was coupled with BSA (PG-204-BSA) (Figure 4) via a diethylene glycol linker to immunize mice for the generation of antimycolactone monoclonal antibodies (mAbs). Their findings suggested that upper-side modification along with the mycolactone core constitute part of the epitope of mAbs. In this direction, more effective protein-coupled analogues targeting mycolactone toxin through antibody adjuvant therapy can be explored.^80−82^

#### Opportunities in the Mycolactone Biosynthesis Machinery

These significant changes affect the biological activity of the resulting mycolactone molecules. Therefore, we propose that genetically engineered mycolactone PKS modules provide a multitude of opportunities in developing intermediate mimics or complex small molecules as potential mycolactone biosynthesis inhibitors. This could be by targeting potential enzymes of the PKS enzyme assembly line, i.e., TE, ACP, KS, or KR.

**Figure 5:**
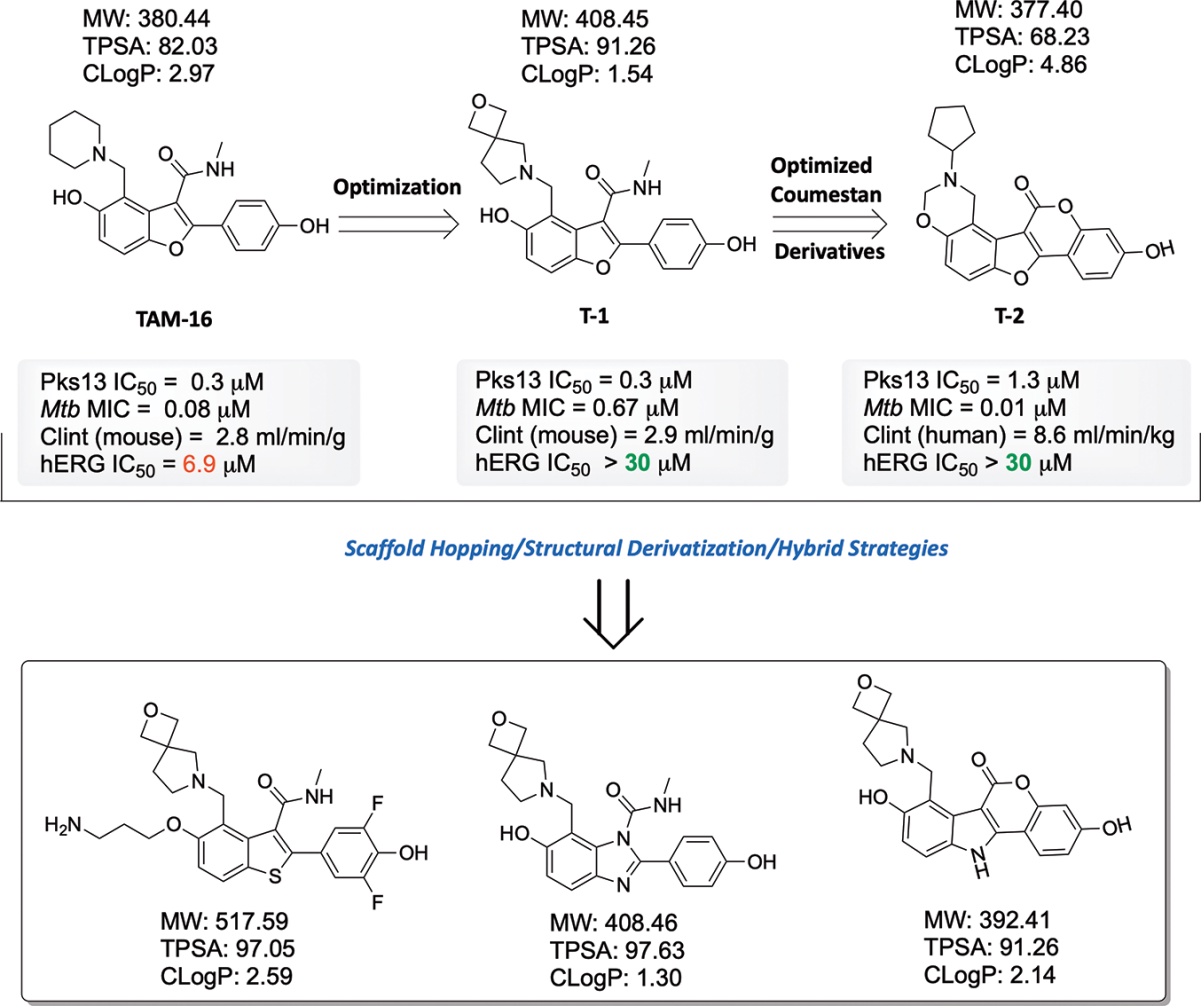
Modular thioesterase inhibitor TAM16 was identified as a potent candidate against *M. tuberculosis*, though its hERG toxicity brings challenges toward clinical approval. TAM16 was further optimized to analogue T-1 and T-2, which resulted in improved hERG toxicity. MW, molecular weight; TPSA, total polar surface area. CLogP: logarithm of the partition coefficient (measure of lipophilicity). In this context, by utilizing scaffold hopping, structural derivatization, and hybrid strategies, a conclusive summary of further developments to accelerate drug discovery is highlighted.

**Figure 6:**
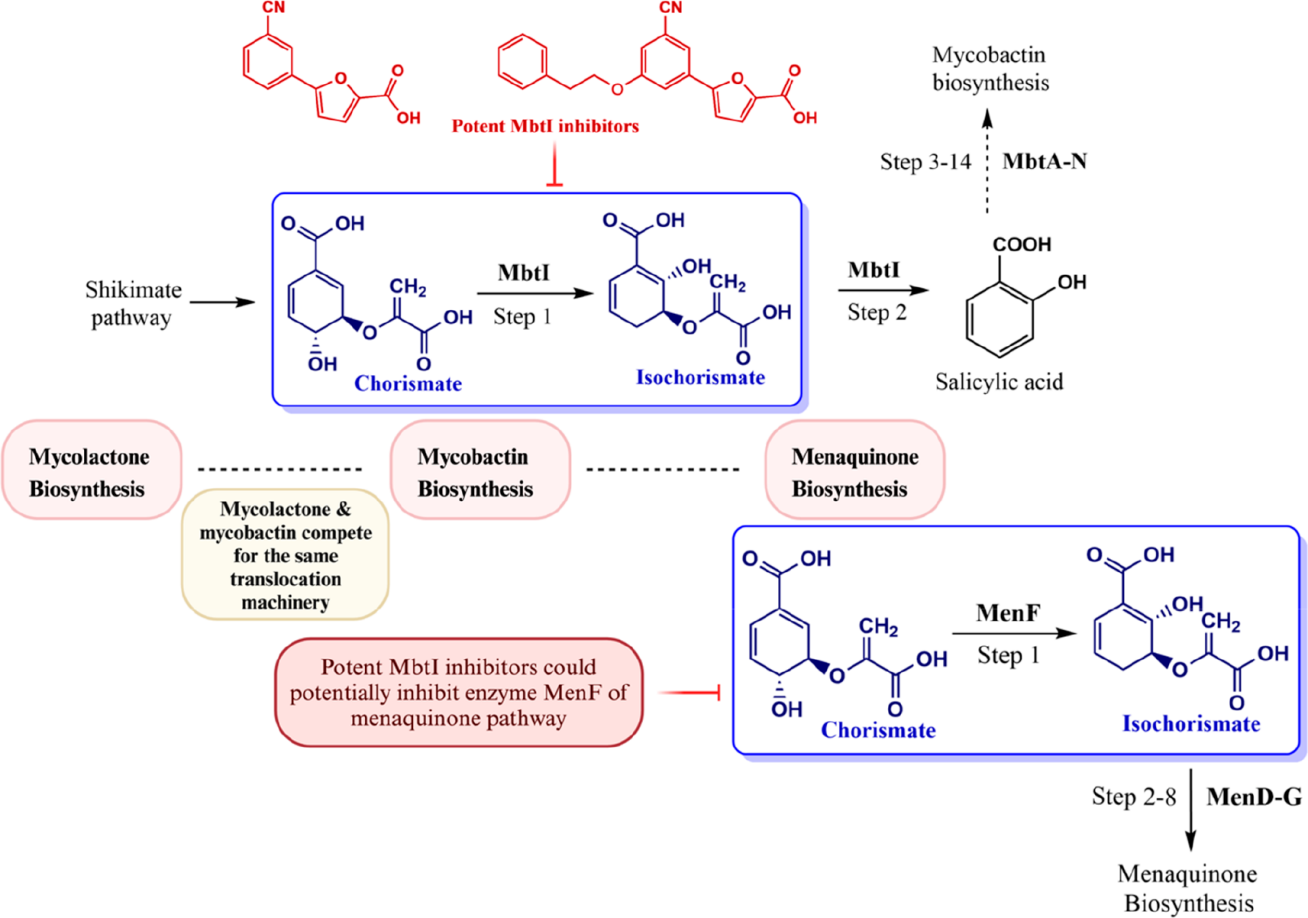
3M mechanisms (mycolactone, mycobactin, and menaquinone biosynthesis pathways) of mycobacteria to invade the mycobacterial antimicrobial defense as an alternative and immediate solution. Implementing established MbtI inhibitors to target unexplored MenF of the menaquinone pathway might be an interesting attempt at drug discovery.

1. Repurposing and reframing the previously identified benzofuran-based analogues including TB thioesterase (TE) inhibitors, such as TAM16 (benzofuran analogue), a clinical trial candidate (analogue of TAM1), and coumestan derivatives, against *M. ulcerans* and *M. leprae* would be a significant attempt.^83−85^ Scaffold hopping/hybrid strategies and structural derivatization on benzofuran analogues along with their balanced physiochemical properties and low hERG cardiotoxicity discussed here (Figure 5) could be a potential move to encounter challenges associated with TAM16. Another strategy can be targeting the intermediate products generated by the specific clusters like *mlsA* or *mlsB.* The available X-ray crystallography structures of the B1-type ACP domain from module 7 of *mlsB* (PDB ID: 6H0Q)^86^ and the A1-type ACP domain from module 5 of *mlsA1* (PDB ID: 6H0J)^87^ provide an opportunity to accelerate drug discovery.
2. Pks13 (Rv3800c), a type I polyketide synthase, has received significant interest recently from the TB community as a viable therapeutic target. In human mycobacterial diseases that are caused by *M. tuberculosis*, *M. leprae*, and *M. ulcerans*, Pks13 is one of the virulence factors and is involved in the final biosynthesis step of mycolic acids.^88,89,12^ The biosynthesis of polyketides typically involves a variety of PKS domains, but the AT, ACP, and KS domains, along with phosphopantetheinyl transferase (PptT), form the core of all PKSs and are necessary for the elongation of the starting unit (Figure 6).^90,91^ The therapeutic potential of coumestan analogues as potent Pks13 inhibitors in the treatment of TB has been explored.^92−94^ Benzofuran, thiophenes, and phenylindole scaffolds have also demonstrated strong Pks13 inhibition.^83,95,96^ As PptT inhibitors, amidinoureas offer a fresh perspective for the immediate repurposing of amidinoureas against the rising HD and BU load.^97^
3. Mycolactone biosynthesis is tightly regulated by the sigma (σ) subunit of RNA polymerase, termed SigA-like promoter.^86^ It can be interesting to target SigA to tackle *M. ulcerans*; however, there is no crystal structure available. Homologous modeling or artificial intelligence-based AlphaFold protein structure predictions could serve the primary purpose, and the ZINC database could potentially help in hit identification from high-throughput scaffold hopping.^98−100^ However, one should be cautious in targeting σ factors, as they hold both potential benefits and risks. The main benefits can be, but are not limited to, the following: (1) yielding broad-spectrum antimicrobials, as σ factors are ubiquitous and highly conserved among bacteria, making them promising targets for broad-spectrum antibiotics, and (2) reduced antibiotic resistance, as σ factors are not directly involved in antibiotic resistance mechanisms, so targeting them is less likely to drive the emergence of resistant strains compared to conventional antibiotics. The critical risks include (1) disrupting the essential cellular processes of beneficial bacteria and harming them and (2) the potential for toxicity via interfering with the fundamental transcription machinery (or off-target metabolic machinery) of host cells. Toward this end, the discovery of a spiro-heterocyclic compound, GPI0363, that inhibits the transcription of *Staphylococcus aureus* via the primary σ factor of RNA polymerase, SigA, is a good example of supporting the above discussion. GPI0363 shares no cross-resistance with other clinically used RNA polymerase inhibitors, such as rifampicin and fidaxomicin.^101^ Overall, targeting σ factors has the potential to revolutionize antimicrobial therapy by providing broad-spectrum, resistance-sparing antibiotics. However, careful consideration of the potential risks and off-target effects is essential to ensure the safety and efficacy of such drugs.

#### Novel Drug Conjugate Approach As an Alternative Solution: Discovery of P218-Mycolactone Conjugates Utilizing Click Chemistry Approach

Recently, the discovery of P218 as a potential *M. ulcerans* DHFR enzyme inhibitor was reported. In this context, shedding light on P218 conjugation with mycolactone utilizing a “Trojan Horse”-like approach against BU could be an interesting attempt to investigate.^102,103^ Blanchard and co-workers have explored the total synthesis of mycolactone analogues.^104^ It has been observed that C-8 methyl substituents and C12′, C13′, and C15′ are central for understanding key interactions of mycolactone and exploring their modes of action. Based on this assumption, the click chemistry approach can be utilized to synthesize P218-mycolactone conjugates, which could be further evaluated against *M. ulcerans* (Figure 4). Here, a rationally designed P218-mycolactone conjugate development strategy is proposed, and further mycobacteriologists may accelerate the investigation of this conjugate against *M. ulcerans*.

However, targeting virulence factors like mycolactone instead of essential cellular pathways could have a direct impact on a bacterium’s viability and growth, such as disruption of pathogenesis (where virulence factors are crucial for a bacterium’s ability to invade and colonize a host, evade the immune system, and cause disease), reduced host damage (where virulence factors are often responsible for causing damage to host cells and tissues, as in the case of BU), and selective pressure for resistance (it may indirectly exert selective pressure, driving the development of antibiotic resistance). The last example may lead to the compensatory mechanism by evolution, although these are yet to be evaluated in studies of BU. Nonetheless, targeting virulence factors offers a more nuanced approach to antimicrobial therapy, aiming to disarm bacteria rather than outright killing them. This strategy has the potential to reduce the emergence of antibiotic resistance, minimize host damage, and preserve the beneficial microbiome, making it a promising alternative to traditional antibiotic treatment.

### An Uncanny Resemblance between Mycolactone and Mycobactin Metabolism

The study reported by Deshayes et al. drew an interesting correlation that the decrease in mycolactone is linked to siderophore-mediated iron-uptake upregulation.^105^

#### Siderophores and Their Conditionally Essential Functions in Mycobacteria

Like other mycobacteria, *M. ulcerans* and *M. leprae* require iron for growth, while high extracellular iron levels could cause irreparable oxidative damage.^106,107^ Iron acts as an enzyme cofactor in several essential biological processes (respiration, DNA synthesis, and protection from reactive oxygen species),^108^ and due to this essentiality, the host also restricts access to iron as an antimicrobial mechanism. In the iron-limiting macrophage milieu, mycobacteria have evolved a specific iron-scavenging mechanism to acquire iron from the host environment. Particularly in iron-stress conditions, mycobacteria secrete conditionally essential iron scavengers: siderophores.^106,107^ Researchers^105^ have highlighted that other than siderophore expression genes (inversely related to IdeR activation), while non-IdeR-dependent proteins involved in iron metabolism are overproduced, such as Mul_3902 (encoded IrtA), siderophore transporters Mul_1209 and Mul_1210, respectively, encoding EsxG and ESsxH, and MmpLS5 and MmpL5 export genes are also overexpressed in 7.5% glucose-enriched medium. They reported that the export machines of mycolactone and mycobactin act as scaffolds for their respective biosynthetic machines, although coupled elongation and export machines are not very uncommon in mycobacteria (it has been noted in the regulation of phthiocerol dimycocerosates and glycopeptidolipids in mycobacteria as well).^109,110^ In correlation, it was hypothesized that mycolactone and mycobactin and their respective biosynthetic complexes compete for the same translocation machinery, which may explain the disappearance of mycolactone when mycobactin production is induced. Henceforth, impeding this identical iron-regulatory mechanism present in both *M. ulcerans* and *M. leprae* could serve as a possible alternative mode of targeting these pathogens. Potential and well-established mycobactin biosynthesis inhibitors such as predominantly discovered MbtA and MbtI inhibitors might also be an alternative treatment option for these neglected mycobacterial strains and could potentially be investigated as repurposing drug candidates.^105,106^

### Mycobactin versus Menaquinone Biosynthesis Pathways

The menaquinone (MK) biosynthesis pathway, present in mycobacteria, plays a crucial role in oxidative phosphorylation and enables both replicating and nonreplicating persisters of mycobacteria to survive under aerobic and anaerobic conditions.^107^ We have drawn intriguing parallels between essential-mechanism menaquinone regulation and conditionally essential-mechanism mycobactin biosynthesis regulation. Although the final product of these pathways differs, the common link between both pathways is the conversion of the starting unit chorismate into isochorismate (Figure 6).^111,105,106^ This enzymatic activity is carried out by the enzyme MbtI (salicylate synthase)^105,106^ in the mycobactin biosynthesis route, whereas the enzyme MenF performs the same function in the MK pathway. Despite several noteworthy studies on the creation of inhibitors for the MenD, MenE, MenB, MenA, and MenG enzymes,^112^ to the best of our knowledge, no inhibitors have yet been reported against MenF. MenF is a crucial enzyme in the Men enzyme assembly; it also oversees the initial steps of MK biosynthesis, and the identification of a potential inhibitor could be considered as a significant addition in antimycobacterial drug discovery research.

In this regard, we draw attention to MbtI inhibitors in investigating activity against MenF and anticipate that the spatial geometry of MenF will accommodate these inhibitors (Figure 6). Over the past 10 years, Chiarelli, Mori, and co-workers have showcased an outstanding amount of research,^113,114,105^ and some top candidates have emerged as prospective MbtI inhibitors. By focusing on two fatal pathways, a sneak peek at these key targets is provided in the hopes that they will be further considered against these underrepresented deadly mycobacterial species.

### Drug Repurposing

#### QcrB

**Figure 7:**
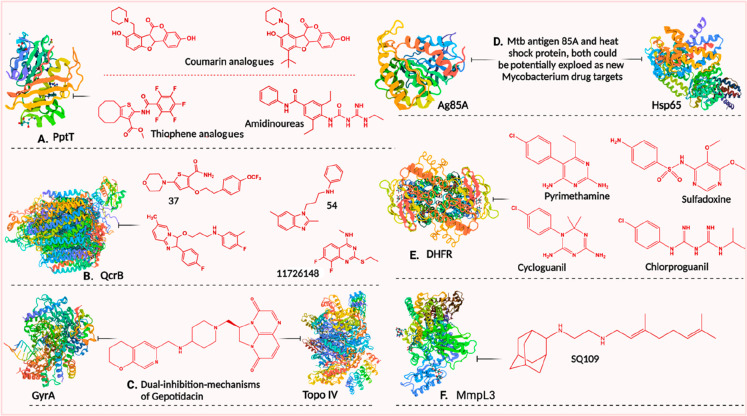
A summary of possible drug candidates with respective drug targets highlighted to accelerate the therapeutic options for NTD treatment: PptT, phosphopantetheinyl transferase; QcrB, *b* subunit of cytochrome *bcc*; GyrA, DNA gyrase A; Topo IV, topoisomerase IV; Ag85A, antigen 85A; Hsp65, heat-shock protein 65; DHFR, dihydrofolate reductase; and MmpL3, mycobacterial membrane protein large-3.

Targeting the membrane-associated *b* subunit of cytochrome *bcc* (QcrB) of the electron transport chain (ETC) has led to the discovery of novel chemically diverse scaffolds, such as Q203 and TB47, which have been very useful for the treatment of these neglected pathogens. Furthermore, to accelerate the investigation of other potent QcrB inhibitors against NTDs, a conclusive summary of a few diverse chemical classes of QcrB inhibitors is highlighted here (Figure 7).^115^

#### DNA Gyrase and Topoisomerase IV

The newest antibiotic in the group, gepotidacin (GSK2140944), was discovered in 2010 by targeting DNA gyrase and topoisomerase mechanisms.^116^ To the best of our knowledge, no other currently licensed antibiotics target DNA gyrase (GyrA) and topoisomerase IV (topo IV) mechanisms in combination. Therefore, this antibiotic’s “dual death mechanisms of action” (Figure 7) make gepotidacin an intriguing clinical drug candidate.^117^ This candidate has demonstrated a remarkable therapeutic window after being tested against a panel of mycobacterial strains, from pathogenic to NTM^116^ mycobacteria strains, which may motivate further research against *M. ulcerans* and *M. leprae*. It would be intriguing to pay attention to the drug candidate’s development against mycobacterial infections over time.

#### Ag85A and Hsp65 Proteins

A discussion on the potentiality of Hsp65 and antigen 85A (Ag85A) DNA vaccines to combat BU has been drawn earlier in the Drug and Vaccine Discovery: How It Started subsection. In accordance, we highlight the potential of target-based drug design and discovery for these two proteins. The discovery of novel drugs can be sped up by utilizing the structure of the *M. tuberculosis* antigen 85A protein (PDB ID: 1SFR).^118^ Additionally, further attempts at homology modeling/artificial intelligence (AI)-based approaches for the *M. ulcerans* antigen 85 protein provide better accuracy. With the help of the crystal structure of *hsp* chaperonin 60.2 (PDB ID: 1SJP),^119^ a comparable potential structure might be generated against HD (Figure 7).

#### DHFR

Compounds of the dihydrofolate reductase (DHFR) class are seen as interesting possibilities against HD and BU. The DHFR inhibitors P218 and epiroprim, as well as K-130 and brodimoprim, have already been discussed earlier (see the New Chemical Entities subsection). In light of this, additional research into alternative DHFR clinical trial candidates, including pyrimethamine, cycloguanil, and chlorproguanil might be interesting to investigate further in the pursuit of a potential cure for HD and BU (Figure 7).^120^

### Essential Drug Targets in Combination with Efflux Pump Inhibitors

**Figure 8:**
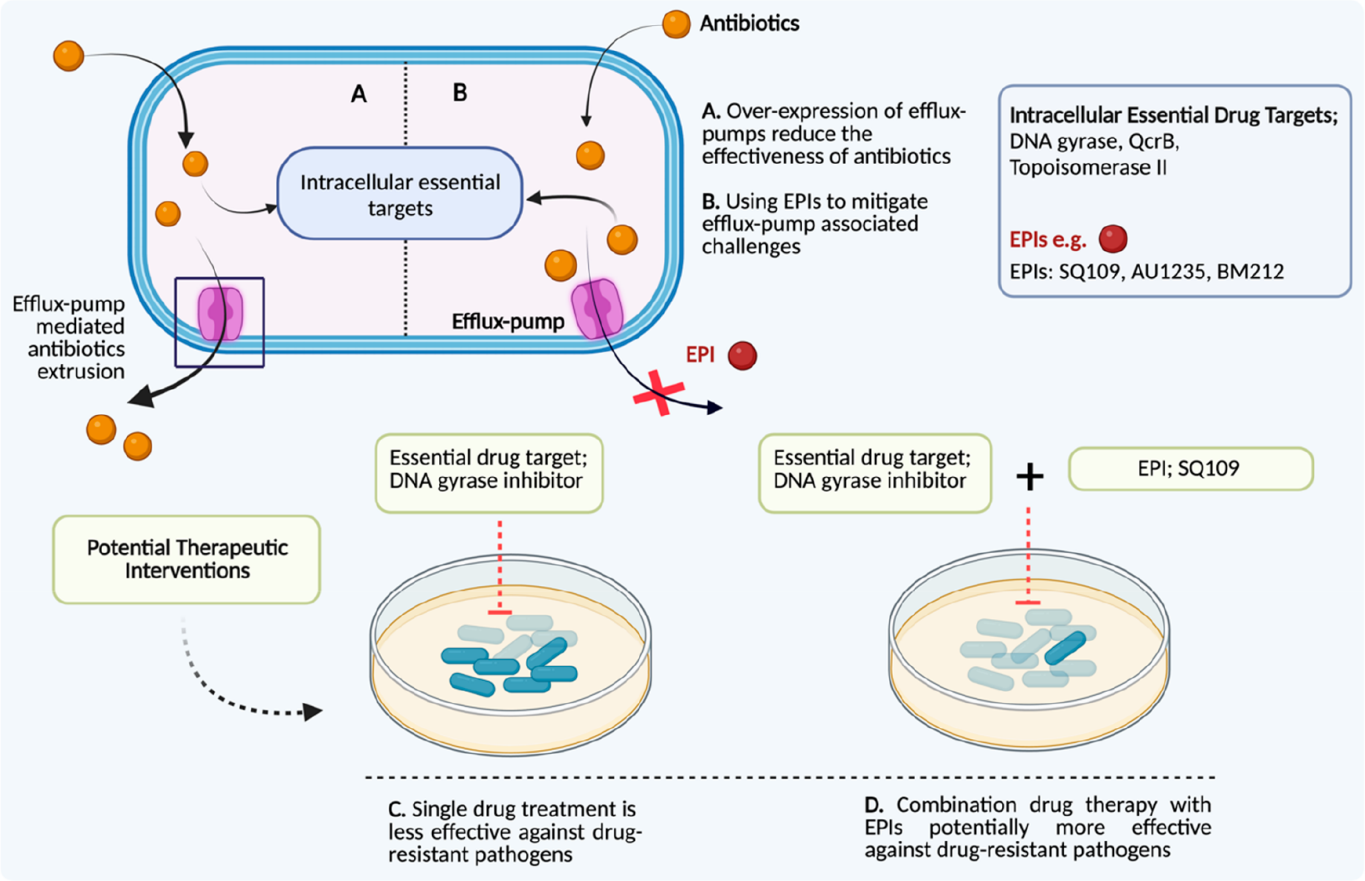
Essential target inhibitors and efflux pump inhibitors (EPIs). Combination drug treatment is required to treat these mycobacterial infections, and in this context, using EPIs could be beneficial with respect to treatment efficacy and treatment duration (leading to shorter treatments). Strategic use of established MmpL3 efflux pump inhibitors such as SQ109, AU1235, or BM212 in combination with other antibiotics could be a game changer against these neglected mycobacterial pathogens.

The emerging drug-resistance burden necessitates novel combination therapeutic approaches for therapeutic interventions, and in this context, efflux pump inhibitors (EPIs) might act as a possible adjuvant in combination therapy.^121^ There are five major classes of efflux pumps, i.e., the ATP-binding cassette (ABC) superfamily, the resistance-nodulation-cell-division superfamily (RND), the small multidrug resistance family (SMR), the major facilitator superfamily (MFS), and the multidrug and toxic compound extrusion family (MATE), contributing to the ever-increasing antibiotic resistance in mycobacteria through antibiotic extrusion from bacterial cytoplasm. Efflux pumps in *M. tuberculosis* and their inhibition to tackle antimicrobial resistance have been discussed elsewhere.^122^ For example, it has been reported that MAV_3306 and MAV_1406, which encode a putative ABC transporter and MFS efflux pump genes, are responsible for extreme azithromycin resistance. Orthologs of efflux pumps encoded by MAV_3306 and MAV_1406 were identified in *M. leprae* and *M. ulcerans*, along with *M. tuberculosis*, *Mycobacterium abscessus*, and *M. marinum* species.^123^ In this context, employing carbonyl cyanide *m*-chlorophenylhydrazone and reserpine (Tap protein-mediated tetracycline extrusion inhibitors; Tap, an EPI of the MFS, is present in *Mycobacterium fortuitum*, conferring resistance to tetracycline and aminoglycoside)^124^ or the promising newly identified EPIs SQ109, AU1235, and BM212 (mycobacterial membrane protein large-3 EPIs) can be promising.^125−127^ The mycobacterial membrane protein large-3 (MmpL3) transporter is essential and required for shuttling the lipid trehalose monomycolate (TMM), a precursor of mycolic acid (MA)-containing trehalose dimycolate (TDM) and mycolyl arabinogalactan peptidoglycan (mAGP), in *Mycobacterium* species.^128^ The mycobacterial cell wall component remains a vital point in mycobacterial drug efflux mechanisms (such as MmpL3)^129^ as well as virulence factor occurrence (FabH-like type III ketosynthase *mup038*(56) within the mycobacterial cell wall). Henceforth, accounting for the growing concern of drug-resistant strains, the strategic use of EPIs in combination with inhibitors of essential mechanisms (DNA gyrase, topoisomerase II, or QcrB, etc.) could inhibit the bacterial growth due to the potential synergistic lethal effect against *M. ulcerans* and *M. leprae*.^130,131^ There are several opportunities to scrutinize how well-established antimycobacterial drugs interact with EPIs, which could be a more beneficial discovery for treating these notorious mycobacterial pathogens (Figures 7 and 8).

### Tailoring Scherr’s Work, Proposing the Blueprints of New Chemical Entities

**Figure 9:**
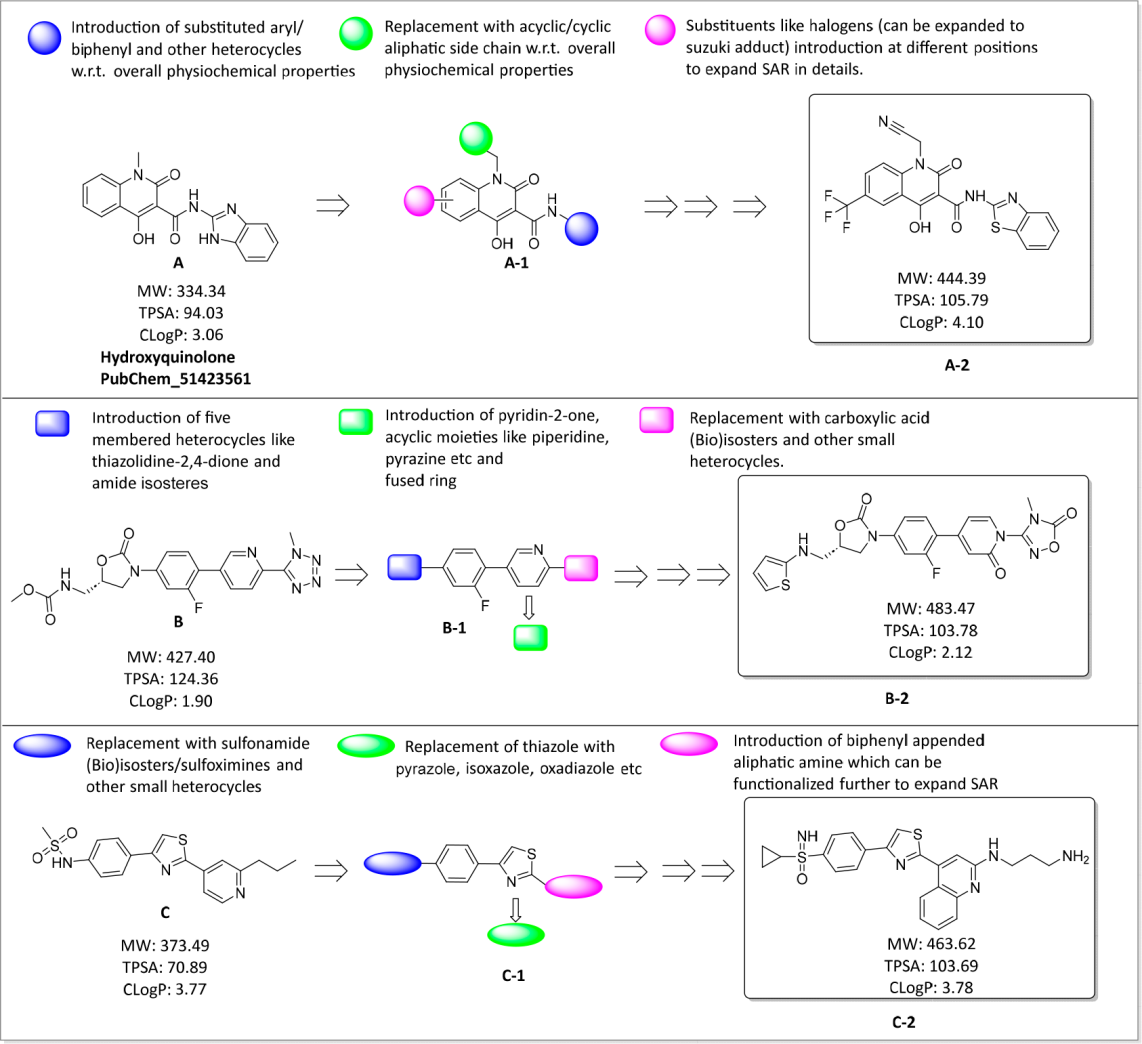
Using Lipinski’s rules, compound structures, i.e., A-2, B-2, and C-2, are proposed to guide medicinal chemistry efforts. Further, A-1, B-1, and C-1 precursor expansion via structural derivatization and scaffold hopping in order to build a comprehensive SAR is demonstrated.

Following the earlier research discussed in the New Chemical Entities subsection, shedding light on heterocyclic rings, medicinal chemists should pay close attention to the pyrazole, thiazole, and tetrazole heterocycles, perhaps by investigating more favorable substitutions in drug design or considering bioisosteric replacements that correspond to Grimm’s hydride displacement rule.^132^ In the same orientation, a hypothetical overview is provided here with a rationale, and a few new chemical entities (NCEs) are proposed for medicinal chemistry investigation. Figure 9 shows the utilization of biorelevant heterocycles, especially those having anti-infectious properties, to design NCEs by maintaining overall good physiochemical properties. The inclusion of different heterocycles and halogen-based substituents in the current design such as thiazolidine-2,4-dione, benzothiazole, oxadiazole, quinoline, and different bioisosteres might impact biological activity against *M. ulcerans.*(133−137) TPSA (total polar surface area) and CLogP (logarithm of the partition coefficient) calculations have been completed using ChemDraw 20.1.1 to demonstrate various correlations between the target molecule and parent molecule. It is further noted that the use of chemoproteomics approaches and different chemical probes has been done to understand the mode of action of the designed molecule and its pharmacological effects.^138^ The medicinal chemistry community could consider these calculations and blueprints to expand qualitative structure–activity relationship (SAR) studies against *M. ulcerans*.

### In Silico Drug Design

Despite the abundance of potential targets, target-based drug discovery has received a lackluster approach for these pathogens. With the use of in silico molecular docking and the incorporation of induced fit docking (IFD), guided molecular dynamics, and MMPBSA analyses for high-throughput screening (HTS), hit identification might be the right direction before laborious chemical synthesis.^139−141^ Undoubtedly, the entire process of drug discovery takes a long time, starting from the initial hypothesis to the development of a candidate that receives clinical approval. In this situation, rather than chemically synthesizing a new candidate, repurposing an existing drug to conduct some preliminary studies against these proteins might be a worthy attempt. Furthermore, by focusing on these accessible protein crystal structures, scaffold hopping could be possible using the HARP (Hansen Disease’s Antimicrobial Resistance Profile) and ZINC databases.^142,100^

Given the high attrition rate, medicinal chemists might make major efforts to find chemical leads through fragment-based drug discovery (FBDD).^143^ FBDD would be useful for finding low-molecular-weight ligands (∼150 Da) that bind to the functionally significant macromolecules listed in Tables 1 and 2. The three-dimensional experimental binding mode of fragments can then primarily characterized by X-ray crystallography, nuclear magnetic resonance (NMR) spectroscopy, or isothermal titration calorimetry (ITC) studies, which aids the optimization of the fragments to potent drug-like lead candidate identification. When comparing the two leading generation methods, HTS and FBDD, FBDD requires less compound screening than HTS and, despite the lower starting potency of the screening hits, produces more effective and lucrative optimization initiatives. To expedite the identification of novel leads for these understudied mycobacterial infections, all of these strategies must be taken into consideration.

**Table 1:** A Conclusive Summary of the Available Protein X-ray Crystal Structures of *M. leprae* in the RCSB PDB

PDB ID	protein crystal information	structure deposited in RCSB PDB
4EX4^144^	the structure of GlcB	2012
4EO9^145^	crystal structure of a phosphoglycerate mutase gpm1	2015
3R2N^145^	crystal structure of cytidine deaminase	2015
4ECP^146^	X-ray crystal structure of inorganic pyrophosphate PPA	2008
3AFQ^147^	crystal structure of the single-stranded-DNA-binding protein (Form II)	2010
3AFP^147^	crystal structure of the single-stranded-DNA-binding protein (Form I)	2010
2CKD^148^	crystal structure of ML2640	2007
2UYO^148^	crystal structure of ML2640C in hexagonal	2007
2UYQ^148^	crystal structure of ML2640C in complex with S-adenosylmethionine	2007
1LEP^149^	three-dimensional structure of the immunodominant heat-shock protein chaperonin-10	1996
2NTV^150^	InhA bound with PTH-NAD adduct	2007
4J07^151^	crystal structure of a probable riboflavin synthase, beta chain RIBH (6,7-dimethyl-8-ribityllumazine synthase, DMRL synthase, lumazine synthase)	2013

**Table 2:** A Conclusive Summary of the Available Protein X-ray Crystal Structures of *M. ulcerans* in the RCSB PDB

PDB ID	protein crystal information	structure deposited in RCSB PDB
4I1Y^152^	cysteine synthase	2012
3QHX^153^	cystathionine gamma-synthase MetB (Cgs)	2011
7KM7,^154^ 7KM8,^155^ 7KM9^156^	dihydrofolate reductase (DHFR)	2020
7K6A^157^		
6UWW^158^		2019
7SH5, 7SMZ, 7TLO^159^	steroid binding CYP142 cytochrome P450 from *M. ulcerans* and *M. marinum*	2021
6AQG^160^	lysyl-tRNA synthetase	2017
3TL3^145^	short-chain type dehydrogenase/reductase	2011
4QJL^161^	phosphopantetheinyl transferase MuPPT	2014

## Concluding Remarks and Outlook

Complex pathogens like *M. leprae* and *M. ulcerans*, which have been poorly investigated and have a great deal of unanswered questions, have slowed progress in finding new chemotypes, validating novel drug targets, and developing cutting-edge screening techniques as promising therapeutic options. In addition to the absence of these scientific methodologies, research funding from different health organizations is scarce, and resource opportunities are constrained. Making a new antibiotic that is clinically viable is a laborious, resource-intensive, and time-consuming procedure, according to the history of antibiotic research. Furthermore, resistance will emerge sooner or later. Given that both of these neglected mycobacteria have a phylogenetic relationship with *M. tuberculosis*, it is important to consider whether repurposing the established TB antibiotics could serve as an immediate alternative treatment. Unfortunately, the TB community is making relatively little effort in this area of neglected mycobacterial pathogens. While the majority of TB research groups are focused on combating the extreme drug-resistant rise of TB, only a significant subset of them is steadfastly focusing on these specific public health concerns. The resurgence of drug-resistant TB in the 1990s was majorly driven by a lack of investment and negligence from big pharmaceutical companies, and the WHO proclaimed TB a global health emergency. Dedicated research efforts are required in this neglected mycobacterial infection domain, or else a comparable situation with newly emerging *M. leprae* and *M. ulcerans* drug-resistant strains may occur sooner rather than later. This emphasizes the urgency of taking immediate action to halt the spread of these drug-resistant strains and, in particular, to support The Global Leprosy Strategy 2021–2030 “Towards zero leprosy”, which is being carried out with the goal of the targeted reduction of new patients,^162^ and the BU_LABNET initiatives, which have already been taken up to improve the quality of BU PCR diagnosis by 11 laboratories in the WHO African region;^163^ however, a substantial number of infections are still occurring. BCG vaccination has huge implications for mycobacterial disease research moving forward and public health, while the clinical trial of LepVax indicates that it is safe and immunogenic in healthy subjects, which is very encouraging for its clinical development.^52^

In light of the grim reality linked to the idiosyncratic nature of mycobacterial strains with rapid genetic evolution and confrontational mutations at the drug targets, great collaborative networking and the persistent involvement of medicinal chemist, computational biologist, mycobacteriologist, and structural biologist groups are needed to achieve the desired goals within a certain time frame. Following this, the participation of translational scientists (safety), pharmacometricians, and clinicians, who contribute crucial components to any discovery team, namely, absorption, distribution, metabolism, and excretion (ADME), pharmacokinetics-pharmacodynamics (PK/PD), and translation to the clinic, is also needed. Then, pragmatic factors related to what will work for the patient population in terms of the desired medication profile and setting up new developmental approaches with long-term safe and efficient chemotherapeutics for the global population need to be determined.

A few strategies could be taken into account. (1) Intermediate mimic development by medicinal chemists by targeting potential metabolic pathways using structure-based drug design (by targeting the mycolactone biosynthesis pathway) in the quest for a new lead candidate identification process. Additionally, by using the principle of bioisosteric substitution, medicinal chemists could lead the optimization for sub- or inactive candidates. To expedite the discovery of antibiotics and lessen the shortage in antibiotic regimens, scaffold hopping using the HARP database, FBDD, or HTS should be more negotiated, as discovering novel chemical entities requires a lot of work. (2) Immunobiologists or mycobacteriologists could potentially investigate the newly designed and chemically synthesized candidates in a meticulous molecular-microbiological sieve. Because there are many factors included in biological investigation approaches, such as excellent MIC, nontoxic profiling, and, more importantly, a compound’s hydrophobic membrane penetration efficiency to kill intracellular survival mycobacteria, parallelly, they can give some thought to evaluating standard TB drugs against these pathogens. Rather than single antibiotic therapy, combination drug therapy might be excellent—“learning from the experiences.” As it has been noted, bacteria are prone to developing antibiotic resistance sooner or later; hence, an initiative to start one step ahead with combination drug therapy, including the use of efflux pump inhibitors, could be a checkmate against these manipulative bacterial devils. Using TB47 or P218 drugs with SQ109 or AU1235 could be a game changer in therapeutic intervention. Even though it is increasingly important more than ever to comprehend how these strains are transmitted through genetic markers or modulations, identifying these genetic markers might be a potential therapeutic intervention at the early stage of these infections. (3) Lastly, the structural biology community should make more investments to produce more protein structures via traditional protein biochemistry approaches or use more contemporary AI technology to anticipate the structures of those metabolically important proteins that are challenging to express. Metabolomics strategies might be used to analyze the orphan enzymes’ ambiguous functions and could be a different way out in the same direction. All of these new therapeutic approaches might revamp new hope for neglected mycobacterial infections.
